# Invasion Dynamics of White-Nose Syndrome Fungus, Midwestern United States, 2012–2014 

**DOI:** 10.3201/eid2106.150123

**Published:** 2015-06

**Authors:** Kate E. Langwig, Joseph R. Hoyt, Katy L. Parise, Joe Kath, Dan Kirk, Winifred F. Frick, Jeffrey T. Foster, A. Marm Kilpatrick

**Affiliations:** University of California, Santa Cruz, California, USA (K.E. Langwig, J.R. Hoyt, W.F. Frick, A.M. Kilpatrick);; Northern Arizona University, Flagstaff, Arizona, USA (K.L. Parise, J.T. Foster);; Illinois Department of Natural Resources, Springfield, Illinois, USA (J. Kath, D. Kirk);; University of New Hampshire, Durham, New Hampshire, USA (J.T. Foster)

**Keywords:** fungal disease, invasive species, Myotis lucifugus, disease invasion, environmental reservoir, fungi, bats, white-nose syndrome, Pseudogymnoascus destructans, United States

## Abstract

White-nose syndrome has devastated bat populations in eastern North America. In Midwestern United States, prevalence increased quickly in the first year of invasion (2012–13) but with low population declines. In the second year (2013–14), environmental contamination led to earlier infection and high population declines. Interventions must be implemented before or soon after fungal invasion to prevent population collapse.

Invasion of novel wildlife diseases has caused widespread declines or species extinction among birds, amphibians, and mammals (*1*–*4*). White-nose syndrome (WNS), caused by the fungal pathogen *Pseudogymnoascus destructans*, is a recently emerged disease of hibernating bats (*5*) that has caused substantial declines in 6 species; bats of 2 species are predicted to become globally extinct (*3*). In little brown bats (*Myotis lucifugus*), tissue damage from fungal infection results in a cascade of physiologic disruptions resulting in death 70–100 days after infection (*6*).

Although the seasonal dynamics of *P. destructans* were recently characterized (*7*), the dynamics of *P. destructans* invasion of new sites has yet to be described. In the 2 years since the identification of *P. destructans*, the extent of the population decline differed each year and among species for unknown reasons (*3*). Furthermore, the role of *P. destructans* in the environment remains unclear (*8*) because no study has reported co-occurring patterns of *P. destructans* in bats and on substrates. We hypothesized that yearly differences in death rates result from changes in the timing of infection as *P. destructans* becomes established and that the environment serves as a source of infection for bats (bats that leave summer maternity sites are not infected; *7*).

## The Study

To test our hypothesis, we studied the invasion dynamics of the WNS fungus by sampling bats of 5 species at 2 hibernacula in central Illinois, USA. We collected samples twice each winter for 2 years (2012–13 and 2013–14). The hibernacula were moderately sized (5–10 hectares, 2–5 m high) abandoned limestone mines that bats use for fall mating and hibernation from September through April. During each visit, we counted all visible bats at each site, which produced complete census data for 4 of the 5 species. Accurately collecting census data for bats of the remaining species (*Eptesicus fuscus*) was difficult because these bats, unlike those of other species, roosted primarily behind crumbling slabs of rock around mine entrances, which were dangerous and difficult to survey.

During each site visit we sampled 15–20 bats of each species by epidermal swabbing (*7*). We also sampled the wall or ceiling of hibernacula under, near (10–20 cm), and far from (>2 m) roosting bats by using the same swabbing technique. Samples were tested for *P. destructans* by using real-time PCR (*9*); according to a serial dilution experiment, the limit of detection was ≈50 conidia.

**Figure 1:**
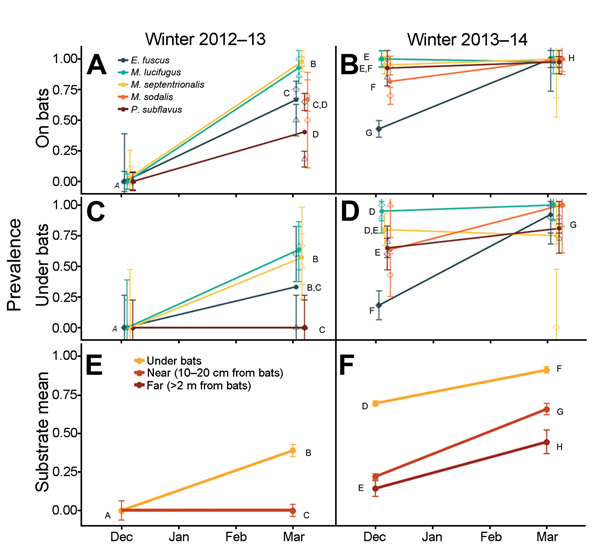
*Pseudogymnoascus destructans* prevalence (±1 SE, calculated from the variance of a binomial distribution sample) over 2 winters, 2012–13 and 2013–14, at 2 sites (diamonds and triangles) in Illinois, USA, on bats of 5 species (A, B); prevalence of *P. destructans* on substrate under bats of each species (C, D), and prevalence of *P. destructans* under, near (10–20 cm), and far from (>2 m) bats (E, F). No substrate samples far from bats were taken in the first winter. Lines join observed mean prevalence for each species (solid circles) to facilitate presentation but do not indicate trajectories between time points. Prevalence of species or substrate means indicated by the same letter did not differ significantly (p>0.05) in a logistic regression analysis with either species and site as fixed effects at each sampling point (A, B) or substrate sample type at each sampling point (C–F); effect of site was not significant in any of these comparisons. *E.*, *Eptesicus*; *M.*, *myotis*; *P*., *perimyotis*.

We obtained 611 samples from bats and 444 from substrate. In early winter of 2012–13, only 1 individual (*Myotis septentrionalis*) of 129 bats of 5 species sampled was positive for *P. destructans*, and none of the 46 substrate samples were positive (Figure 1, panels A, C, E). Just 4 months later, in March 2013, prevalence was >85% for bats of 2 species (*M. septentrionalis, M. lucifugus*), 40%–75% for 2 species (*E. fuscus*, *Myotis sodalis*), and 15%–60% for 1 species (*Perimyotis subflavus*) at the 2 sites (Figure 1, panel A). The prevalence of *P. destructans* on the substrate under these bats varied from 0% to 67%, and substrate prevalence paralleled fungal prevalence for bats of each species (Figure 1, panel C). Despite widespread apparent infection of bats at this time, none of the 36 substrate samples taken just 10–20 cm from bats were positive for *P. destructans* (Figure 1, panel E).

In early winter of the next year (late November 2013), patterns differed markedly from those of the previous early winter. *P. destructans* was already widespread in the environment, found in 70% of samples from under bats, 22% of samples 10–20 cm from bats, and 14% of samples >2 m from bats (Figure 1, panels D, F). Prevalence among bats of 4 species was already >70%, and prevalence among bats of 1 of these species (*P. subflavus*), for which prevalence at the end of the previous winter had been lowest, was already 85%–100% (Figure 1, panel B). By the end of the second winter, 109 (98%) of 111 bats were positive for *P. destructans*, and *P. destructans* was present throughout the hibernacula (in 91% of samples from under bats, 66% of samples near bats, and 44% of samples far from bats) (Figure 1, panels B,D,F).

**Figure 2:**
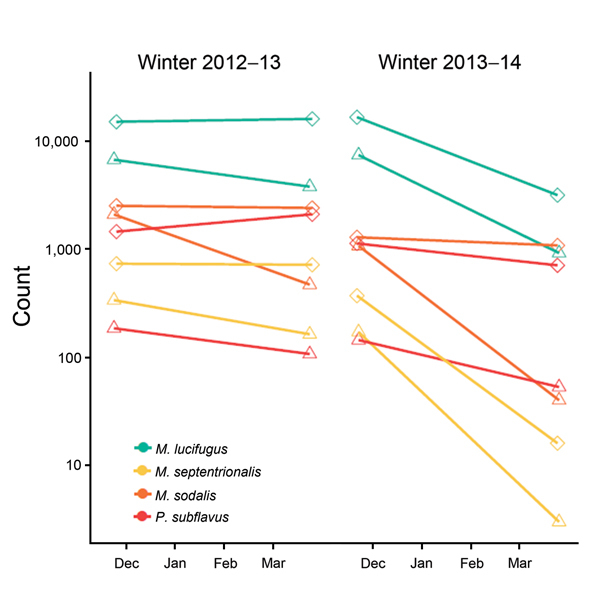
Complete population counts on a log scale of 4 species of bats at 2 sites in Illinois, USA, over 2 winters, 2012–13 and 2013–14. Diamonds and triangles indicate sites.

Over these 2 years, the effect of WNS on bat populations mirrored the patterns of *P. destructans* prevalence. During the first winter, declines were limited at the larger site and moderate (50%–75%) at the smaller site (Figure 2). In contrast, over the second winter, counts of *M. septentrionalis* bats declined by 95%–99% and *M. lucifugus* bats by 81%–88% (20,000 bats of this species disappeared) (Figure 2, panel A). Populations of bats of the 2 other species also experienced moderate to severe declines in the second year (*M. sodalis*, 16%–96%; *P. subflavus*, 47%–73%) (Figure 2, panel B). Declines probably resulted from disease-related deaths because high hibernacula site fidelity makes emigration unlikely (*10*) and substantial numbers of dead bats were observed at both sites.

## Conclusions

Early in the first winter studied, prevalence of *P. destructans* was very low, and although transmission resulted in most bats harboring *P. destructans* by winters’ end, declines in bat populations were limited. In contrast, early in the second winter, fungal prevalence among bats was already high and severe communitywide declines occurred over the next 4 months. The earlier timing of exposure in the second year would be expected to increase the effects of WNS because by winter’s end most bats would have been infected and in hibernation for at least 70–100 days (the approximate time between infection and death; *5*). Few would be able to survive until spring, when bats cease hibernating and clear the fungus (*7*).

Patterns of *P. destructans* distribution in the environment mirrored prevalence among bats and population declines. Early in the first year, when *P. destructans* was rare on hibernacula substrates, most bats were not infected in early winter, and 4 months later, *P. destructans* was not detectable in one third of bats of 3 species. However, by the end of the first winter, *P. destructans* was present on hibernacula substrate under bats, probably resulting from bats shedding *P. destructans* into the environment. At the beginning of the following winter, *P. destructans* was widespread in the environment, and nearly all bats had fungus on them. The widespread occurrence of *P. destructans* in the environment at this time may have contributed to higher prevalence among bats because most bats clear infections during the summer, when their body temperature is too high for *P. destructans* growth (*7*,*11*). Long-term persistence of *P. destructans* in the absence of bats (*8*,*12*) suggests that an environmental reservoir of *P. destructans* may contribute to WNS persistence, as occurs for other diseases, such as cholera (*13*).

WNS continues to spread south, west, and north from New York, where it was first detected in 2006, and continues to cause widespread bat population declines. Potential control strategies include development of probiotic treatments (*14*) and alteration of hibernacula microclimates to make them cooler and drier (*3*,*15*). Our results suggest that if *P. destructans* invasion in other sites is similar to what we documented in Illinois, interventions must be implemented proactively, or quickly after *P. destructans* invasion, to prevent collapse of bat communities. Reduced bat populations will probably have a negative effect on humans because bats play a useful role in ecosystems by consuming disease vectors and many forest and agricultural insect pests.
